# Implicit Standardization in a Minority Language Community: Real-Time Syntactic Change among Hasidic Yiddish Writers

**DOI:** 10.3389/frai.2020.00035

**Published:** 2020-05-29

**Authors:** Isaac L. Bleaman

**Affiliations:** Department of Linguistics, University of California, Berkeley, Berkeley, CA, United States

**Keywords:** corpus sociolinguistics, minority languages, syntactic variation, particle verbs, standardization, Yiddish, Hasidic Jews

## Abstract

The recent turn to “big data” from social media corpora has enabled sociolinguists to investigate patterns of language variation and change at unprecedented scales. However, research in this paradigm has been slow to address variable phenomena in minority languages, where data scarcity and the absence of computational tools (e.g., taggers, parsers) often present significant barriers to entry. This article analyzes socio-syntactic variation in one minority language variety, Hasidic Yiddish, focusing on a variable for which tokens can be identified in raw text using purely morphological criteria. In non-finite particle verbs, the overt tense marker *tsu* (cf. English *to*, German *zu*) is variably realized either between the preverbal particle and verb (e.g., *oyf-tsu-es-n* up-to-eat-INF ‘to eat up’; the conservative variant) or before both elements (*tsu oyf-es-n* to up-eat-INF; the innovative variant). Nearly 38,000 tokens of non-finite particle verbs were extracted from the popular Hasidic Yiddish discussion forum *Kave Shtiebel* (the ‘coffee room’; kaveshtiebel.com). A mixed-effects regression analysis reveals that despite a forum-wide favoring effect for the innovative variant, users favor the conservative variant the longer their accounts remain open and active. This process of rapid implicit standardization is supported by ethnographic evidence highlighting the spread of language norms among Hasidic writers on the internet, most of whom did not have the opportunity to express themselves in written Yiddish prior to the advent of social media.

## 1. Introduction

In recent years, sociolinguists have increasingly turned to social media platforms like Twitter to investigate large-scale patterns of language variation and change. Some of the areas that have been addressed include gender and style (Bamman et al., 2014), the geographic diffusion of lexical variants (Eisenstein et al., 2014; Huang et al., 2016; Grieve et al., 2018), and the grammatical and social constraints on orthographic variation (Eisenstein, 2015). Social media corpora have increased not only the number of speakers (or writers) whose data can be analyzed in a single research project, but also the range of variables that can be effectively studied: in a corpus containing tens of millions or even billions of words, one can uncover robust sociolinguistic patterns even for variables that occur with low frequency in conversational interviews.

While the field of sociolinguistics continues to gain valuable insights from “big data” in social media, most of this work contributes to our understanding of only a handful of language varieties—American English chief among them. The research bias favoring monolingual majority communities has been a longstanding problem in sociolinguistics (Meyerhoff and Nagy, 2008; Stanford, 2016; Guy and Adli, 2019), and it certainly extends to social media studies. Unfortunately, many of the existing tools in computational linguistics (including stemmers, part-of-speech taggers, and syntactic parsers) were not designed to support minority language data. Even if raw text data can be obtained—which is not always the case, especially for endangered varieties—the lack of computational tools to process the data presents fundamental challenges to large-scale research on these languages and their users. This may explain why social media studies of minority languages, including Welsh (Jones et al., 2013), Māori (Keegan et al., 2015), Limburgish, and Frisian (Nguyen et al., 2015), tend to focus on macro-level social phenomena such as language choice rather than micro-level linguistic phenomena such as grammatical variation.

One minority language that has been considered exemplary of “resource-poor” languages is Yiddish (Genzel et al., 2009), which is spoken at home by some 170,000 Americans, 86% of whom reside in New York State (U.S. Census Bureau, 2015). According to the engineers who developed Google Translate in Yiddish, the reason for this designation is the problem of data scarcity: the lack of large parallel corpora makes it difficult to obtain the training data necessary for automatic machine translation. They argue that if engineers can overcome these challenges for Yiddish, they would be well-positioned to address similar challenges in other “low-resource” languages—“a very important public service that will help preserve these languages and make literature in these languages available to the rest of the world” (Genzel et al., 2009, p. 6).

Ironically, the availability of Google Translate in Yiddish has led to the proliferation of fake Yiddish websites, thus exacerbating the problem of data scarcity for other applications. For example, students interested in the usage of particular words and phrases must now sift through pages of search results containing both reliable Yiddish-language sources, including newspaper articles, and unreliable ones, including blogs whose authors used Google Translate to render their posts in many different languages, presumably to increase reader traffic^1^. For linguists interested in the grammar of minority languages, including Yiddish, the ubiquity of machine-translated text raises serious questions about the reliability of data taken from the internet. For example, software like BootCaT (Baroni and Bernardini, 2004), which builds corpora by scraping the web for pages containing target-language keywords, inadvertently includes some of these machine-translated websites. Fortunately, recent years have also seen an increase in the number of *real* Yiddish websites, including discussion forums designed for Hasidic Jews who make up the vast majority of today's native speakers.

The goal of this article is to show not only that a corpus study using online Hasidic Yiddish is feasible, but also that it can yield novel findings about linguistic variation comparable to those obtained from social media studies of majority languages like English. The current study analyzes socio-syntactic variation on a popular Hasidic Yiddish discussion forum, focusing on particle verbs and the relative position of the non-finite tense marker *tsu* ‘to.’ Tokens of this variable can be identified in raw text using purely morphological criteria, without the need for a part-of-speech tagger, a parser, or even a dictionary, none of which have yet been developed for Hasidic Yiddish. In addition to linguistic constraints on the variable, the study uncovers a significant social fact: although the discussion forum shows a modest increase in the probability of the innovative variant, users favor the conservative variant the longer their accounts remain open and active. This finding, framed as an example of *rapid implicit standardization* on the internet, is supported by ethnographic evidence highlighting the role of the discussion forum in spreading language norms among its Hasidic Jewish users.

This study has important consequences for the analysis of variation in minority languages, as it demonstrates the utility of computational methods even for a language variety, Hasidic Yiddish, without an extensive online presence or linguistically processed corpora of any size^2^. Given that majority languages including English are actually *over-represented* on large social media platforms like Twitter (Mocanu et al., 2013), it is especially encouraging that smaller discussion forums can provide adequate minority language data for variationist sociolinguistics. This study also contributes to our understanding of contemporary Hasidic Yiddish, which has been overshadowed in linguistic research by projects focused on the European dialects spoken before the Holocaust (Nove, 2018). The results of this study corroborate the view—one taken for granted by sociolinguists but still uncommon among specialists in Yiddish studies—that seemingly inconsistent and disorderly linguistic behavior among Hasidic Jews is in fact principled and orderly, conditioned by linguistic and extra-linguistic factors in predictable ways.

The article is organized as follows. Section 2 introduces the online community (the discussion forum *Kave Shtiebel*) from which a sociolinguistic corpus was built for this study. Evidence will be presented to show that these anonymous writers are Hasidic Jews who reside primarily in New York. Section 3 introduces the syntactic variable, which has not previously been mentioned in linguistic descriptions of Yiddish; for this reason, most of the hypotheses about quantitative constraints (presented in 3.2) are drawn from studies of particle verb phrases in English, which involve a different set of variants. Section 4 describes the method for automatically extracting tokens of the variable from the forum's posts. Section 5 presents the results of the statistical analysis of the variation, laying out the relevant constraints and their interpretations. This section also offers a detailed discussion of two seemingly contradictory effects relating to real-time syntactic change among forum users (presented in 5.2). Finally, section 6 summarizes the conclusions and the questions they raise for future sociolinguistic studies of minority language corpora.

## 2. The Corpus and the Community

For the religiously conservative Hasidic community, the maintenance of a Jewish vernacular language reflects a broader ideology that opposes acculturation to non-Jewish norms (Isaacs, 1999). Hasidic Jews in the United States constitute an urban speech community, as they are geographically concentrated in a few Yiddish-speaking neighborhoods in Brooklyn and Upstate New York. Yiddish is used as a medium of instruction in private Hasidic schools, which are segregated by gender and feature very different curricula in terms of both content and language. Boys receive an essentially monolingual education in Yiddish; English is only taught from third to eighth grade (approximately age 7–13), and during those years, it is only taught for ninety minutes a day in the very late afternoon, a period reserved for all non-religious subjects. Girls, by contrast, have a fully bilingual curriculum from first grade through the end of high school, with Yiddish used for religious subjects and English for secular subjects (Fader, 2009, pp. 22–23). The imbalance in bilingual proficiency between men and women has been cited by community members as one reason why Yiddish-language discussion forums tend to be men's spaces. By contrast, the most popular forum among Hasidic women, imamother.com, is written in English.

While the Hasidic community is committed to the maintenance of Yiddish, its leaders do not support efforts to standardize the language. The use of Yiddish is strictly enforced in Hasidic schools, but subjects like “grammar” (norms of language use) and “composition” (writing skills) are viewed as distractions from serious religious study and are not emphasized in Hasidic curricula. Hasidic Jews have played virtually no role in the standardization efforts of secular organizations like the YIVO and the League for Yiddish, and Hasidic publishers have never endorsed their standards. This is not to say that Hasidic Jews lack standard language ideologies; as mentioned below in section 3, Hasidic consultants agree that in non-finite particle verbs, one variant often sounds “more correct” than the other. The language ideologies of Hasidic men and women are discussed in more depth in Bleaman, 2018.

Universal literacy in Yiddish means that Hasidic newspapers and magazines enjoy sizable readerships, but very few Hasidic adults have a regular need to write in Yiddish after finishing school. This was articulated to me offline in a sociolinguistic interview I conducted with Berl (33 years old; Monsey, NY), who works as a freelance writer. (All names of interviewees are pseudonyms.)

It used to be, until… literally ten or fifteen years ago, if a person wasn't a Yiddish writer and he wasn't studying in *koylel* [religious school for married men] where he'd have to write down his ideas about the Torah or take notes… there literally wasn't, that kind of person didn't have to write a single sentence in Yiddish in twenty years. There was nowhere to write, no reason to write, nobody to write for. At work he'd write in English, obviously, nobody writes in Yiddish at work. His grocery list is English. He just didn't write. Zero.

(Translated from Yiddish.)

Berl's reference to “ten or fifteen years ago” alludes to the advent of Hasidic blogs, and later of online discussion forums and WhatsApp groups specifically for Hasidic users—all of which have afforded community members new opportunities to express themselves in written Yiddish. The role of the internet in rejuvenating Hasidic writing was articulated in many of the sociolinguistic interviews I conducted with Hasidic Jews offline (Bleaman, 2018). Another Hasidic man, Duvid (36; Monsey), told me that before participating in *Kave Shtiebel*'s poetry competition he had never done any creative writing whatsoever, in Yiddish or any other language.

Hasidic discussion forums have existed since at least 2005. In that year, a now-defunct Hebrew-language forum called *Hyde Park* had a Yiddish-language subforum called *heymishe shtusim* ‘Hasidic nonsense.’ The subforum was designed as a place where Yiddish-speaking Hasidic men could post their questions and concerns related to sexual matters (masturbation, premature ejaculation, marital relations) which are considered taboo to discuss publicly. Over time, writers began to discuss other more mundane topics, including sports, which are also seen as inappropriate for Hasidic Jews. In 2006, a standalone forum called *iVelt* (short for *idishe velt* ‘Jewish world’; ivelt.com/forum) was launched, which has since become increasingly mainstream in its ultra-Orthodox religious and social outlook.

A second independent forum, *Kave Shtiebel* (kaveshtiebel.com), was launched in February 2012. Its name refers to the ‘coffee room’ of a study or prayer house, where men can take a break and chat casually over a cup of coffee. *Kave Shtiebel* (KS) was founded in response to mounting frustration with the moderation of *iVelt*, where posts that were critical of Hasidic power structures (especially the authority of the rabbis) were routinely deleted. KS prides itself on giving writers the freedom to post socially critical content, alongside other topics including history, science, religion, politics, and poetry. This commitment is codified in its guidelines for new members. In recent years, KS users have also come together to publish an *offline* magazine, with original content touching on religious and secular topics. This magazine, *Veker* ‘lit., one who awakens,’ is sold on Amazon and at newsstands in Brooklyn and other neighborhoods.

Because the users of Hasidic discussion forums are largely inexperienced amateur writers—having attended schools where writing skills are not developed systematically—there is understandably a significant amount of variation in the written Yiddish found on the internet today, including orthographic inconsistencies. At the same time, one might expect the overall amount of variation to decrease over time, as writers develop their skills and acquire norms from one another. Indeed, there is anecdotal evidence suggesting this trend. A lively conversation ensued in response to a message I recently posted to KS (November 10, 2019) soliciting specific examples of writing conventions that users have acquired since joining the forum. The responses mentioned norms in spelling and punctuation, such as the difference between a comma and a period. One user, writing under the username *Gefilte fish*, identified the singular role that KS has played in his development as a writer:

[My experience on] *Kave Shtiebel* taught me not only how to write in Yiddish, spelling, grammar, but I couldn't even use the Hebrew keyboard before I got here. Here I've learned how to spell in Yiddish, including the difference between *in* and *and*, and many other things that I can't recall at the moment. Go back to my first posts from 2012 and you'll see that I spelled like a grandma. (Grandmas, don't take it personally. You write very well. I mean no disrespect, it's just a turn of phrase.) […] Of my graduating class in *yeshiva* [religious school] I couldn't name even three people who can write a “sentence” (*zats*?) in any language, not Yiddish, not English, not Hebrew.

(Translated from Yiddish.)

*Gefilte fish's* inexperience as a writer prior to joining KS is indicated by his having acquired the ability to type in Hebrew (Yiddish is written using Hebrew characters) and the orthographic distinction between two basic function words (*in* and *and*, which are spelled differently in Hasidic publications but are homophonous in the Central Yiddish dialect used by Hasidic Jews: [ɪn]). The quote also suggests that his development as a writer is ongoing: he questions whether *zats* is the correct Yiddish word for ‘sentence,’ which he initially presents as an English borrowing in Hebrew characters.

Another user, *Katle kanye*^3^, wrote that whenever he isn't sure which spelling or vocabulary variant to use, he types the options into KS's search box to compare their relative frequencies. If neither variant is more common than the other, he opts for the one used by the KS writers whom he most respects.

The current study provides quantitative support—from one area of Yiddish syntax, non-finite particle verbs—showing that KS writers are shifting toward greater use of normative grammatical features over time as they interact on the forum. This is a process that I term *rapid implicit standardization*, and it will be explicated in the discussion that follows.

### 2.1. The “Coffee Room” and Its Hasidic Writers

The linguistic data for this study come from the Hasidic discussion forum *Kave Shtiebel*. In order to use an online forum to analyze variation in a minority language variety, it is important to establish who its users are and to what speech community they belong offline. The fact that nearly all KS writers are Hasidic men from the greater New York area is clear from the language of the forum itself: KS is written in Yiddish following Hasidic orthographic conventions, and its posts regularly include phrases from rabbinic texts written in Hebrew and Aramaic (which are the core of Hasidic boys' but not girls' education) as well as borrowings from New York English. Not surprisingly, some of the most active threads are concerned with politics and current events in the New York Hasidic community (and satellite towns such as Lakewood, NJ).

KS is extremely protective of users' confidentiality, and users virtually never disclose any personal information in their profiles. Still, it is possible to identify broad demographic trends in the forum's metadata. The founders of KS granted me access to the database containing all public posts, which I downloaded most recently on October 23, 2019. (This same content could have been obtained by scraping the forum's pages.) The corpus, representing approximately seven and a half years of activity, contains 29 million word tokens across 392,660 posts by 2,194 users.

**Figure 1:**
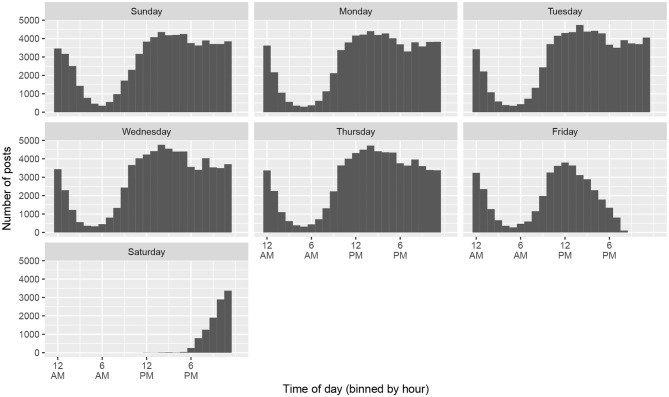
All posts from *Kave Shtiebel* by timestamp and day of the week (Eastern Time Zone).

Figure 1 plots all the posts in the database, grouped by the day of the week on which they were written and binned into hourly intervals (Eastern Time Zone). The figure reveals two important social facts: First, KS writers are concentrated on the East Coast, since there is a daily lull in activity when East Coast residents typically sleep. Second, virtually all KS writers observe the Jewish Sabbath from Friday evening through Saturday evening, when the use of computers and smartphones is prohibited. The expectation that users observe the Sabbath is also mentioned in KS's guidelines for new users. Tellingly, its Yiddish localization of the forum software phpBB translates “Saturday” as *motse-shabes* ‘the evening following the Sabbath,’ which assumes that all posts with a “Saturday” timestamp are written after sunset.

**Figure 2:**
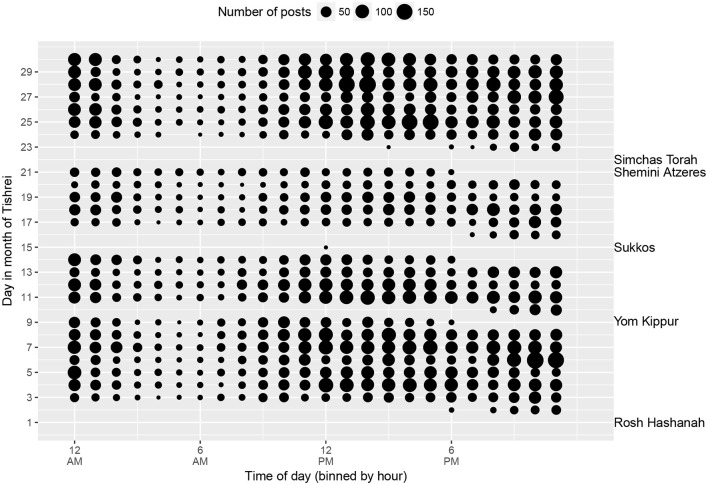
All KS posts written during the Jewish month of Tishrei, 5773-5780 (2012-2019), by time of day (Eastern Time Zone) and day of the month. Jewish holidays when computer use is prohibited are indicated to the right of the plot.

The same trend of Orthodox religious observance is evident from a plot of all posts to KS during the Jewish month of Tishrei, coinciding with parts of September and October (Figure 2). Virtually no messages are posted during the major holidays (Rosh Hashanah, Yom Kippur, etc.) when the use of electronic devices is prohibited.

While the two graphs suggest that KS users are Orthodox Jews on the East Coast, they do not show that users are necessarily Hasidic New Yorkers. The only direct evidence of this comes from offline interactions with KS users. I first joined KS as a way to recruit Hasidic Jews for sociolinguistic interviews as part of a larger research project (Bleaman, 2018). Although my Yiddish recruitment letter did not specify demographic criteria for participation, the 12 KS users I met in person had remarkably uniform social characteristics. All of them were native Yiddish-speaking men, aged 25–36, and affiliated with Hasidic communities—most from the Satmar community, but with some representation from the Vizhnitz and Tosh communities. All of them were living in Hasidic neighborhoods in the New York area (Williamsburg, Boro Park, and Monsey), had attended Hasidic schools for their entire education, had gone through arranged marriages, and were working for Hasidic businesses.

Although this discussion strongly suggests that KS writers belong to the Hasidic Yiddish speech community offline, it would be a mistake to draw any definitive conclusions about “(Hasidic) Yiddish” as a whole based on a study of the forum alone. Doing so would overlook the inherent stylistic differences that exist between spoken and written language, as well as the possibility of internet- or even platform-specific registers of written language. Some research in computational sociolinguistics has found that social media writing approximates certain aspects of speech, such as the high frequency of first- and second-person pronouns compared to third-person pronouns in discussion groups (Yates, 1996, pp. 40–42) and the linguistic constraints on orthographic *t,d*-deletion (e.g., *lef* for *left*) and *g*-deletion (*talkin*) on Twitter (Eisenstein, 2015). However, other studies have shown that online registers make use of features (or rates of features) that diverge from users' spoken repertoires, such the use of African-American English variants by gay white Reddit users from the UK (Ilbury, 2019) or the use of restrictive relative clauses headed by a pronoun (e.g., *we who #FeelTheBern*), which are readily found on Twitter despite being stylistically marked (Conrod et al., 2016). The mixed results of these studies should caution us against extrapolating linguistic patterns in speech from linguistic behavior in writing on the internet.

The comparability of speech and online writing is further complicated for contemporary Yiddish, due to the opposition of Hasidic leadership to online communication. Hasidic rabbis have issued decrees against the use of internet-enabled smartphones (Deutsch, 2009), and Hasidic Jews who require internet access for work are expected to install community-mandated web filters (Fader, 2017). One of the ways this is enforced is that parents must certify in writing that they have installed filters on their phones (making them “kosher”) before they can enroll their children in school. These filters block access to websites that are considered improper for Hasidic visitors; some evidently even block *Kave Shtiebel*, although not *iVelt*. Despite these prohibitions—and as the impetus for these prohibitions—Hasidic Jews are increasingly using the internet for everyday communication and entertainment. Just as Hasidic entrepreneurs have realized the potential of the internet for business (Deutsch, 2009, p. 4), so too have everyday Hasidic consumers become avid users of internet media, circulated on Hasidic websites and in Hasidic WhatsApp groups.

These considerations highlight some of the limitations of KS data. Not only does the forum reflect the online writing of men of a narrow age range, but its users engage in practices that are considered subversive by the standards of the Hasidic community. Still, KS is one of the most well-known Yiddish websites, Hasidic or otherwise, and its members come from the largest community of Yiddish speakers in the United States. There is also no clear evidence suggesting that the language of KS differs radically from written Hasidic Yiddish offline, especially in its grammatical properties. Even if the results of a study of KS cannot directly address language patterns in the wider speech community, they may offer insights which can become the hypotheses for further research.

## 3. Particle Verb Variation in Yiddish

The linguistic focus of this study is a syntactic alternation involving particle verbs in non-finite tense phrases in Yiddish.

Particle verbs (also known as *phrasal verbs*) are combinations of verbs and preposition- or adverb-like particles, which together form a close semantic unit (Dehé, 2015, p. 611). In English, particles invariably appear after the verb (e.g., *throw up, hang out*). In Yiddish, particles appear before the verb in most syntactic contexts. For example, particles always precede the verb in the infinitive, such as when a particle verb phrase appears as the complement of a modal like *must*:


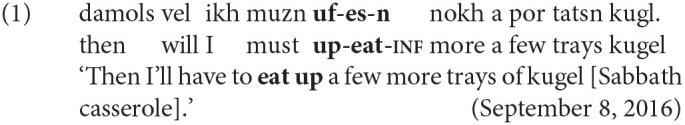


(Note: Yiddish is written in the Hebrew alphabet. All examples from the KS corpus are provided in standard YIVO transliteration. Hyphens have been added to show morpheme boundaries.)

While modals select for bare infinitival verb phrase (VP) complements, other verbal, nominal, and adjectival predicates select for tense phrase (TP) complements. This context licenses an overt non-finite tense marker, *tsu* (a cognate of English *to* and German *zu*), in addition to the infinitival suffix on the verb (-*n*). The contrast between non-finite VP and TP complements is illustrated below in (2) and (3); note that the contrast is also found in English.


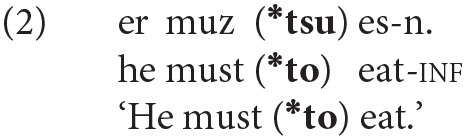



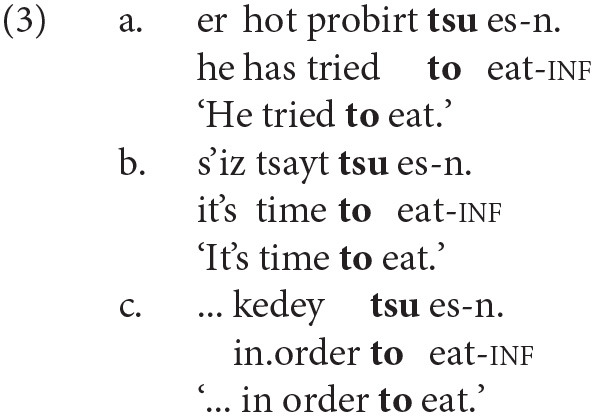


### 3.1. Variable Word Order in Non-finite Particle Verbs

The variation analyzed in this article concerns the relative position of *tsu* ‘to’ in non-finite particle verbs. Generally, *tsu* appears between the preverbal particle and the verb, and the combination is usually written as a single word (e.g., *oyf-****tsu****-es-n* up-**to**-eat-INF ‘to eat up’). However, *tsu* sometimes appears before both the preverbal particle and the verb, usually separated by a space (e.g., ***tsu****oyf-es-n* **to** up-eat-INF). Examples of the two variants are shown below in (4) and in (5). These sets of near-minimal pairs are both from the KS corpus.


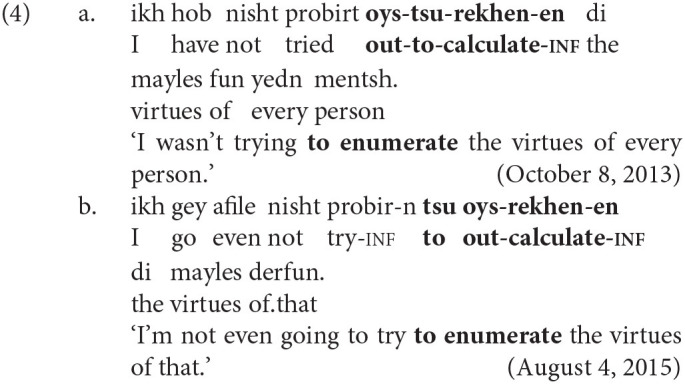



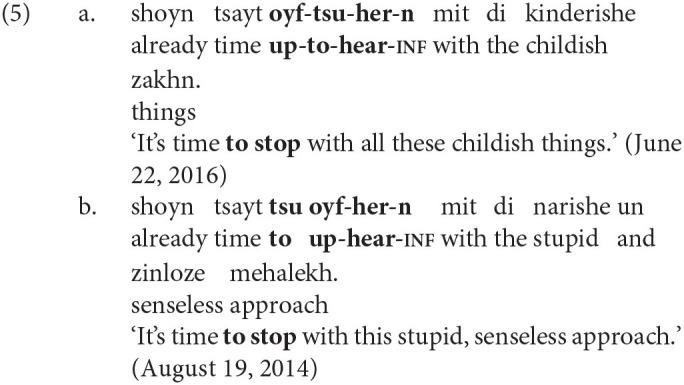


Throughout this article, the label *PtoV* (**P**article-**to**-**V**erb) will be used to refer to the variant in which *tsu* ‘to’ intervenes between the particle and verb, as in (4-a) and (5-a). The label *toPV* (**to P**article-**V**erb) will be used when *tsu* precedes both elements, as in (4-b) and (5-b).

The *PtoV* order is the only possibility mentioned in the Yiddish grammatical literature (Mark, 1978, p. 330; Schaechter, 1995, p. 64) and the only one taught in university-level Yiddish classes. It is also by far the more common variant in contemporary Hasidic Yiddish, as this article will show. The use of *toPV* is very likely to be a change in progress: It is relatively rare in publications printed in pre-Holocaust Eastern Europe^4^ and it is not attested in the dialectological data on the Hasidic community's European source dialects^5^. Many non-Hasidic native speakers of Yiddish judge *toPV* to be totally ungrammatical. Nevertheless, the *toPV* order is readily found in informal Hasidic Yiddish text on the web and is also attested in newer Hasidic publications indexed in Google Books.

As with other proposed syntactic variables, one must ask whether *PtoV* and *toPV* are truly variants of one another—that is, whether they are equivalent either in meaning or in discourse function. The existence of near-minimal pairs like (4) and (5) may be the best evidence of functional equivalence. As a secondary check, three native speakers of Hasidic Yiddish (all *Kave Shtiebel* users) were asked to comment on a number of example sentences. When shown sentences with one variant, native speakers informed me that the other variant would “mean the same thing” (but that *PtoV* often sounded more “correct”). Of course, while these intuitions suggest equivalence, native speakers are likely to be unaware of, or unable to characterize, the various factors that correlate with the use of either variant (see Silverstein, 1981). It is one task of variationist analysis to determine what these factors might be.

Since Yiddish grammars do not mention the *toPV* variant, the factors that affect the use of *PtoV* or *toPV* are not at all understood. Fortunately, the variable lends itself to analysis using a social media corpus like KS, for a few different reasons. First, non-finite particle verbs do not occur very frequently in spoken Yiddish, so a very large corpus is required to obtain the requisite number of tokens for thorough analysis^6^. Second, tokens of the variable can be identified on purely morphological grounds, simply by extracting all strings beginning with a valid Yiddish particle and ending with the infinitival suffix -*n*, with *tsu* appearing either before or after the particle. Using morphological criteria to identify tokens is particularly helpful in the case of Hasidic Yiddish, a minority language variety in which there are no dictionaries or part-of-speech taggers to rely on when searching through raw text.

### 3.2. Particle Verb Variation in English and Predictions for Yiddish

The variable word order of particle verb phrases is among the most well-studied alternations in the syntactic literature. In English, the variation involves the relative ordering of postverbal particles and non-pronominal objects in transitive verb phrases, as shown in (6).


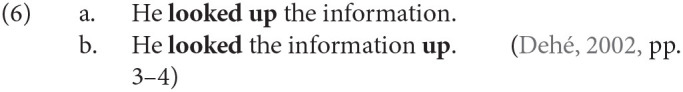


When discussing the variation in English, I follow the convention of Dehé (2002) who uses the term “continuous” to refer to instances when the verb and particle are adjacent (6-a) and “discontinuous” when they are not (6-b).

Although the syntactic alternation in Hasidic Yiddish (pronouncing *tsu* ‘to’ before or after the preverbal particle) differs from the alternation in English (pronouncing the object before or after the postverbal particle), they are superficially similar in that one variant involves strict adjacency between verb and particle while the other does not. In other words, *toPV* could be described as “continuous” because the verb and particle are adjacent, and *PtoV* could be described as “discontinuous” because the verb and particle are separated by *tsu*. For this reason, it is worth considering the literature on particle verb variation in English in order to formulate hypotheses about the variation in Yiddish, which has not been documented before^7^.

In one of the earliest sociolinguistic studies of the alternation, Kroch and Small (1978) identify the “degree of semantic dependence of particle on the verb” as one linguistic predictor of the word order variation. The intuition is that combinations of verb and particle whose meaning cannot be predicted from the sum of their parts (e.g., *throw up* ‘vomit,’ *put up* ‘temporarily house’) function as standalone predicates and are most easily parsed when the verb and particle are adjacent. The idiomaticity of the particle verb combination has been shown in many studies to be among the strongest predictors of the variation, and considerable work has been done to define it formally (see Lohse et al., 2004; Bannard, 2005). The tendency for idiomatic combinations to remain structurally or linearly adjacent is also involved in categorical grammaticality judgments. Zeller (2001, pp. 89–90) observes that German allows for the topicalization of particles when the combination is semantically transparent [e.g., *auf-geh-en* up-go-INF ‘rise’ in (7-a)] but not when it is idiomatic [e.g., *auf-hör-en* up-hear-INF ‘stop’ in (7-b)]. The same judgments hold for English (8) and Yiddish (9).


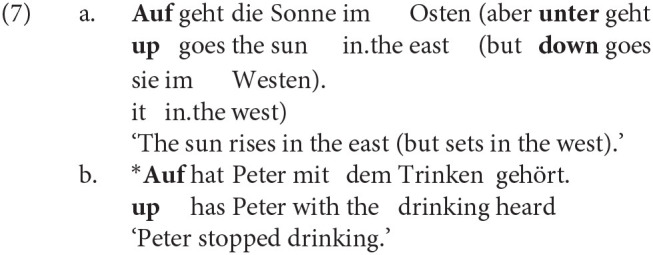



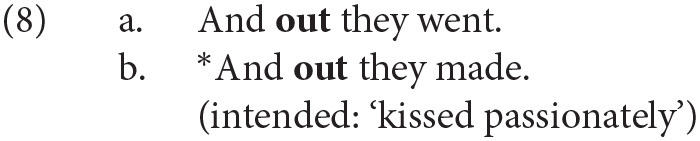



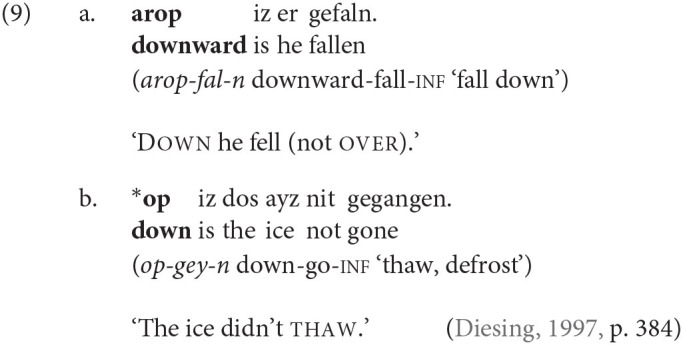


Gries (2001) presents an overview of various factors that linguists have proposed as predictors of the variation in English and offers a unified account based on processing effort/cost: for example, the more morphosyntactically complex an object is (correlated with the number of words it contains), the more difficult or cognitively “expensive” it is to process the discontinuous order. The same holds true of idiomatic particle verb combinations compared to ones that are semantically transparent. If speakers aim to facilitate effective communication by minimizing the processing cost for the listener, then it follows that more complex particle verb phrases (e.g., long idiomatic ones) will favor the continuous order, which is cognitively easier to process (Rohdenburg, 1996). A related proposal has been offered by Lohse et al. (2004), who focus on the size of the processing domain and its relationship to the syntactic and semantic properties of the particle verb construction.

In Yiddish, as in English and other Germanic languages, combinations of particle and verb vary in terms of their semantic transparency or compositionality (Mark, 1978, p. 308; Diesing, 1997, pp. 383–384; Talmy, 2000, p. 297). Directional particles combine with motion verbs to yield semantically transparent combinations (e.g., *aroys-gey-n* outward-walk-INF ‘walk out, exit’). By contrast, non-directional particles combined with the same verbs often have idiomatic meanings (*oys-gey-n* out-walk-INF ‘expire; die’)^8^. If idiomatic combinations prefer to remain adjacent (*toPV*), it could be because they are (variably) derived via the morphological incorporation of the particle into the verb; this would (variably) prevent the intervention of *tsu* between the two elements, just as it prevents the topicalization of the particle (Diesing, 1997, p. 384). Under this theory, these particles would behave (at least some of the time) like genuine prefixes, which are always adjacent to their verbs (*toP*[*refix*]*V*; see Biskup et al., 2011 on prefix and particle verbs in German). Regardless of how semantic transparency is reflected in syntactic derivations, its role will be examined in the current study by means of grouping Yiddish particles into different types, discussed in section 4.2.

Another predictor of the variation in English is the information entropy of the particle, which is used to gauge its productivity or ability to associate with different verbs (Schnoebelen, 2008). Information entropy works in this way: For each particle, we generate a list of all of the unique verbs with which it appears in the corpus, and the number of times it appears with each of those verbs. Entropy is low if a particle only appears with a small number of different verbs, and high if it appears with a variety of verbs at roughly equal rates^9^. It is assumed that particles with low entropy are less productive than high entropy particles. Combinations with low productivity particles may be considered more “wordlike,” and are expected to favor the variant in which the particle and verb are adjacent: the continuous order in English, and *toPV* in Yiddish.

Social factors have also been shown to condition the variation in English. Kroch and Small (1978) demonstrate that talk radio hosts use the continuous order at a significantly higher rate than listeners do when calling into the show. They take this as evidence that the standard language ideology favoring the continuous order is active in everyday linguistic behavior and can serve as a marker of status^10^. Haddican and Johnson (2012) find significant differences between UK/Irish English and North American English, with the latter favoring the continuous order at higher rates than the former in both production (gleaned from Twitter data) and perception (a sentence rating task). They also find that the relative frequency of the discontinuous order has increased over time, based on evidence drawn from a historical corpus.

If standard language ideology promotes the *PtoV* variant in Yiddish, then one might hypothesize a positive correlation between *toPV* and the use of other non-standard features, including non-standard spellings. To test this hypothesis, the analysis below will consider whether there is a non-standard orthographic form anywhere in the non-finite particle verb token (in the particle, in the verb, or in the use of *tsi* for *tsu*, a common spelling variant reflecting the spoken dialect of Hasidic Jews).

Finally, if *toPV* is a change in progress within Hasidic Yiddish, then we also expect younger speakers (and writers) to use the innovative *toPV* variant at higher rates than older speakers (and writers). Unfortunately, KS cannot currently be used to analyze age-based sociolinguistic stratification, because the corpus represents less than eight years of activity (February 2012 through October 2019) and because its writers seem to come mostly from the same generational cohort (married men under 40). However, KS can still be used to study the effect of time, on the forum as a whole and in the posts of individual users. The hypotheses with regard to syntactic change in progress are presented in section 4.2.

## 4. Data Processing and Analysis

### 4.1. Building the Dataset: Extracting Tokens of Non-finite Particle Verbs

On October 23, 2019, the database containing all public posts from KS was downloaded and imported into a data frame, with one column representing the content of the post and other columns containing the post's metadata. Using Python scripts, each message was stripped of HTML tags and text quoted from other users, and then tokenized—i.e., converted from a long text string to a list of individual words, excluding punctuation. Each token was also stripped of all characters not contained in the standard Hebrew alphabet, e.g., apostrophes and diacritics, including those found within pre-combined Unicode characters sometimes used in non-Hasidic Yiddish. Word-final letter forms (*langer nun, shlos-mem*, etc.) were also converted to non-final forms to avoid certain inconsistencies within Hasidic orthography^11^.

At this point, Yiddish grammars (in particular, Mark, 1978, pp. 301–311 and Jacobs, 2005, p. 210) were consulted to generate a list of all Yiddish particles^12^, supplemented by common variants used in Hasidic Yiddish^13^. Posts were then searched for all word strings beginning with any of these particles, followed by *tsu* (or *tsi*, a dialect spelling), and ending with the infinitival suffix, *-n*. In this way, it was possible to rely on morphological criteria to identify particle verbs, rather than a pre-defined dictionary. This yielded a list of 36,370 *potential* examples of *PtoV* non-finite particle verbs, representing 3,704 unique strings.

These potential *PtoV* tokens were used to generate a list of all potential verbs, i.e., just the substring after the particle and *tsu*. This list of potential verbs—containing exactly 1,300 unique strings—was exported to a text file and hand-checked for accuracy^14^. A number of these items were removed because they were not actually verbs^15^, and additional non-standard spellings were added to the list. A script was then used to assemble the full list of all theoretically possible particle verbs, by combining every particle with every (hand-verified) verb. At this point, all KS posts were searched for matches of all non-finite particle verbs appearing in either order: *PtoV* or *toPV*^16^.

This method of using morphological criteria (plus manual verification) to identify non-finite particle verbs yielded 37,858 tokens of either *PtoV* or *toPV*. Of these, 5,553 tokens (14.7%) were of the innovative/non-standard *toPV* variant. This final dataset represents 1,768 unique (spelling-normalized) particle verb combinations from 1,165 users.

### 4.2. Coding Independent Linguistic and Non-linguistic Factors

Each token of the dependent variable (*PtoV* vs. *toPV*) was coded for a variety of potential conditioning factors including social, grammatical, and cognitive predictors (Tamminga et al., 2016). These factors, which were tested in the full regression model, were:

**Categorical fixed effects**
i. particle type (*directional, cognate, other*);
ii. whether the verb is an English borrowing (e.g., *arayn-tsu-****sken****-en* inward-to-scan-INF ‘to scan in’; *aroys-tsu-****sayn****-en* outward-to-sign-INF ‘to sign out’);
iii. whether the post has been “liked” by another user;
iv. whether the token contains a non-standard spelling (of particle, verb, or *tsi* for *tsu*);
v. persistence (the variant used most recently within the same post: *PtoV, toPV*, or *none*);
**Continuous fixed effects**
vi. the information entropy of the particle;
vii. the number of phonological segments in the (spelling-normalized) particle verb combination;
viii. the log frequency of the (spelling-normalized) particle verb combination;
ix. the number of days elapsed from user registration to the current post's timestamp (i.e., the user's seniority);
x. the number of days elapsed from the launch of KS to the current post's timestamp (i.e., the age of the forum);
**Random effects**
xi. writer (username); and
xii. word (spelling-normalized particle verb combination).

The motivation for including some of these factors was presented in section 3.2, along with predictions based on studies of particle verb variation in English. For clarity, the remainder of this subsection will summarize the predictions for all of these factors in order.

The first factor, particle type, is a way to approximate the semantic transparency of the particle verb combination. As noted by Talmy (2000, pp. 297–298), Yiddish particles can be categorized into three distinct types. The first type includes directional particles (e.g., *arayn* ‘inward,’ *aroys* ‘outward,’ *aroyf* ‘upward,’ etc.) that attach freely to all motion verbs, verbs of transfer, etc., and usually contribute a concrete or metaphorical directional reading to the resulting particle verb. Yiddish also has a series of what I call ‘cognate’ particles, which look like the directional particles but without the initial *ar-* (i.e., *ayn, oys, oyf* , etc.). These are often translated into English as prepositions (‘in,’ ‘out,’ ‘up,’ etc.) and their semantic contribution is generally more idiosyncratic (e.g., *oys-gey-n* out-go-INF ‘expire; die’). The remaining Yiddish particles were classified as “other.” Examples of each of the three particle types are shown in Table 1. (Note that my labels “directional,” “cognate,” and “other” correspond to Talmy's (2000, pp. 297–298) terms “long doublet,” “short doublet,” and “singlet”). If particle verbs with directional particles are maximally transparent in meaning, then perhaps speakers/writers will more readily tolerate their separation from the verb by the presence of intervening *tsu* (i.e., *PtoV*)—much in the same way that Yiddish allows for their topicalization to the front of the sentence (Diesing, 1997, p. 384). If particle verbs with cognate particles are the least semantically transparent, then these combinations should favor strict adjacency (*toPV*). Particles in the catchall “other” category should favor neither variant.

**Table 1:** Examples of the three particle types.

**Particle type**	**Example particles**	**Example combination**	**Translation**
directional	*aroys* ‘outward,’ *aroyf* ‘upward’	*aroys-fir-n* outward-lead-INF	‘lead out(side)’
cognate	*oys* ‘out,’ *oyf* ‘up’	*oys-fir-n* out-lead-INF	‘execute; conclude’
other	*mit* ‘with,’ *nokh* ‘after’	*mit-fil-n* with-feel-INF	‘empathize’

The inclusion of binary factors for whether the verb is an English borrowing, whether the post has been “liked” by another user, and whether the token contains a non-standard spelling is meant to capture intuitions about the social nature of the *toPV* variant. If a writer borrows a particular English particle verb (in which *to* always precedes the verb and particle: ***to****sign in*), we might also expect him to use the innovative/non-standard variant in which *tsu* is the first element (*toPV*; ***tsu****arayn-sayn-en*). Posts that receive a positive social evaluation, in the form of a “like” from another user, might correlate with the use of standard grammatical features, like *PtoV*. Finally, the use of a non-standard spelling in the particle verb token might favor the use of the non-standard variant (*toPV*).

**Figure 3:**
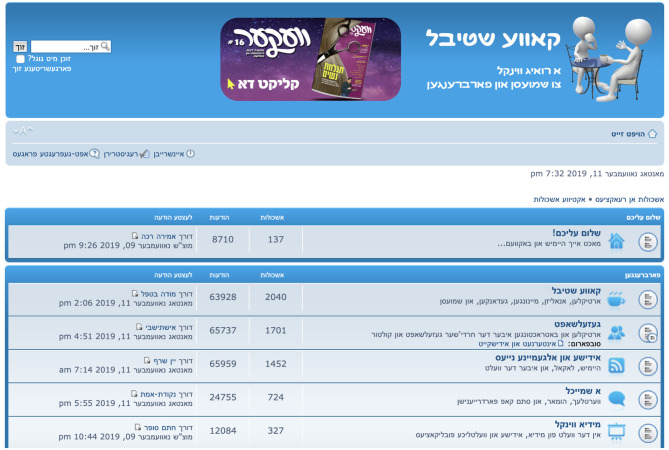
The front page of *Kave Shtiebel* (screenshot from November 11, 2019). Image published with permission of forum moderators.

Persistence describes the tendency for tokens of a recently produced variant to influence subsequent tokens of the variable (Scherre, 2001; Tamminga, 2016; see also Weiner and Labov, 1983, p. 47). Some of the effect is due to the fact that the initial token is “drawn from the same distribution” as subsequent tokens (Tamminga, 2016, p. 343), i.e., from the same speaker, who may be biased to produce one variant at a higher or lower rate than the population mean. However, persistence has been found to be significant even in regression models with random effects for speaker, suggesting a more general cognitive basis (Tamminga, 2016). Although persistence is most relevant in spontaneous speech, it has been found to be a significant predictor of particle verb variation even in written corpora (Gries, 2005). Because KS is designed to be a place for casual anonymous conversation (*a ruig vinkl tsu shmuesn* ‘a relaxed spot to converse,’ as its masthead states; see Figure 3), some of the cognitive constraints on speech production may be preserved in this genre of informal writing, as well. Persistence was captured in this study by means of a discrete variable coded for the most recently used variant within the same post (*PtoV, toPV*, or *none* if the current token is the first of its post). If writers are biased to repeat tokens within posts, then a previous occurrence of *PtoV* should favor the repetition of *PtoV, toPV* should favor repetition of *toPV*, and the first or only token in a particular post (*none*) should not favor either variant.

The information entropy of the particle is meant to capture its productivity. If a particle appears rather predictably only with a small number of different verbs (i.e., low information entropy), the resulting combinations may be more “wordlike” and thus likelier to remain adjacent (*toPV*). Particles with high information entropy attach to a greater variety of different verbs, and the resulting combinations may be less “wordlike” and easier to separate (*PtoV*).

The analysis also includes a factor for the number of phonological segments in the (spelling-normalized) particle verb combination. When KS writers were asked to provide judgments on *PtoV*~*toPV* minimal pairs, some remarked that inserting *tsu* between the particle and verb would make the word “too long” or unwieldy to write and read. Since *PtoV* is usually written as one word but *toPV* as two (i.e., *to PV*), longer particle verb combinations might favor *toPV* merely by virtue of their being longer strings. This hypothesis isn't motivated by existing literature, but rather by users speaking from their personal experience typing on their computers and smartphones. (Note that the number of phonological segments in the string usually coincides with the number of orthographic characters.)

It has been argued in the literature on exemplar models of linguistic knowledge that frequency of occurrence affects the way forms are cognitively stored and produced (e.g., Bybee, 2002). However, the role of frequency in constraining syntactic variation (as opposed to phonological variation) has not been consistent across studies. Some evidence suggests that high lexical frequency can amplify the effects of other constraints but may not have an independent effect of its own (Erker and Guy, 2012). However, attempts at replication have found that constraint effects may actually be stronger for *lower* frequency items (Bayley et al., 2013). The working hypothesis for this study is that since *PtoV* is the overwhelmingly preferred variant (all else being equal), more frequent combinations of particle and verb are likelier to have a larger sheer number of *PtoV* tokens than *toPV* tokens, and therefore a more robust representation of *PtoV* exemplars stored in speakers' episodic memory. Consequently, it is predicted that higher frequency particle verb combinations will favor *PtoV*. Since no standalone corpora of Hasidic Yiddish exist, frequency information for each particle verb combination was calculated from within the generated dataset of non-finite particle verb tokens. Frequency was based on spelling-normalized combinations of particle and verb, to abstract over any typographical differences in raw tokens. Table 2 shows the most frequent combinations in the dataset.

**Table 2:** The five most frequent particle verb combinations from the dataset containing all non-finite particle verb tokens.

**Particle verb combo**.	**Gloss**	**Translation**	**Frequency**
*aroys-breng-en*	outward-bring-INF	‘bring out; express’	868
*on-kum-en*	on-come-INF	‘arrive’	833
*on-nem-en*	on-take-INF	‘accept’	751
*on-heyb-n*	on-lift-INF	‘start’	618
*arayn-gey-n*	inward-go-INF	‘walk in, enter’	583

The number of days elapsed since user registration (i.e., a given user's seniority on KS at the time of the post) and the number of days elapsed since the launch of KS (i.e., the age of the forum at the time of the post) are meant to capture syntactic change in progress. If users are implicitly acquiring grammatical norms over time as they write and engage with other KS members, there should be a positive correlation between user seniority and the use of *PtoV*. If *toPV* is innovative, then we might expect to find a higher probability of *toPV* over time on the forum as a whole, irrespective of any tendency for individual writers to become more standard. Such an effect, if found, should be very modest, since there is no reason to believe that the user demographics of KS (including age) have shifted much from 2012 to 2019.

Finally, the model includes random intercepts for writer (username) and dictionary word (spelling-normalized particle verb combination), as well as by-writer random slopes for all predictors of interest. The inclusion of random effects is important to account for the inherent variability across individual writers and words. For example, some KS users are also professional writers and editors, and they may inherently favor *PtoV* more than other users, show less sensitivity to word length, etc. There will also inevitably be certain particle verb combinations (such as *op-deyt-n*, which is the English borrowing ‘update’) that have an atypical baseline rate for the variable (*tsu op-deyt-n* ‘to update’ is used much more often than *op-tsu-deyt-n*, although both are found in the corpus). Including random effects in the statistical model controls for some of these inherent differences.

## 5. Results

### 5.1. Statistical Analysis

The variation in word order (*PtoV* vs. *toPV*) across all 37,858 non-finite particle verb tokens was modeled through logistic mixed-effects regression using the R package lme4 (version 1.1-17; Bates et al., 2015). The fixed effects included in the full model were the factors numbered (i) through (x) in the previous section. All continuous predictors were standardized. The model also included random intercepts for writer (1,165 different usernames) and for word (1,768 different particle verb combinations), and by-writer random slopes (uncorrelated) for all fixed effect terms.

The model's fixed effects are summarized in Table 3. *P*-values were calculated based on asymptotic Wald tests. The McFadden's pseudo r^2^ for this model was 0.259. Note that a more parsimonious model, excluding all non-significant fixed effects and corresponding random slopes, had very similar coefficients and *z*-values for all the significant predictors.

**Table 3:** Estimates for fixed effects from logistic regression model of variable order in non-finite particle verbs (*n* = 37,858), where positive estimates favor the *toPV* variant; significance codes: *** = <0.001, ** = <0.01, * = <0.05, . = <0.1.

	**Estimate**	**Std. error**	***z*-value**	***p*-value**		***N***
(Intercept)	−2.04	0.10	−19.84	<0.001	***	37,858
Particle type (vs. other)						10,496
cognate	0.60	0.11	5.72	<0.001	***	16,307
directional	−0.52	0.11	−4.79	<0.001	***	11,055
Verb is English borrowing (vs. no)						37,401
yes	0.54	0.19	2.88	0.004	**	457
Post has been “liked” (vs. no)						13,146
yes	−0.07	0.05	−1.63	0.104		24,712
Contains non-standard spelling (vs. no)						30,988
yes	−0.05	0.07	−0.77	0.444		6,870
Persistence (prev. token in post) (vs. none)						26,622
*PtoV*	−0.53	0.06	−9.15	<0.001	***	9,749
*toPV*	0.61	0.07	8.27	<0.001	***	1,487
Particle entropy (scaled)	−0.33	0.04	−7.90	<0.001	***	37,858
Num. segments in particle verb (scaled)	0.07	0.04	1.84	0.066	·	37,858
Log frequency of particle verb (scaled)	−0.10	0.03	−3.11	0.002	**	37,858
Days since user registration (scaled)	−0.13	0.06	−2.20	0.028	*	37,858
Days since KS launch (scaled)	0.28	0.06	4.80	<0.001	***	37,858

Significant main effects (at *p* < 0.05) were found for all but three of the predictors tested: (i) whether the post has been “liked” by another user; (ii) whether the token contains a non-standard spelling; and (iii) the number of phonological segments in the token. Their non-significance is not entirely surprising: (i) KS users seem to “like” posts because of their content, not because of grammatical properties (such as a writer's use of *PtoV*) of which readers may not be consciously aware. (ii) Tokens that were marked as containing a non-standard spelling also included typographical errors, which should have no direct relation to a writer's use of grammatical features. Finally, (iii) although some writers hypothesized that *PtoV* might be disfavored by a general orthographic preference against very long words, the effect for the length of the particle verb (PV) combination, if any, is rather weak.

#### 5.1.1. Effects and Interpretations of Significant Continuous Predictors

**Figure 4:**
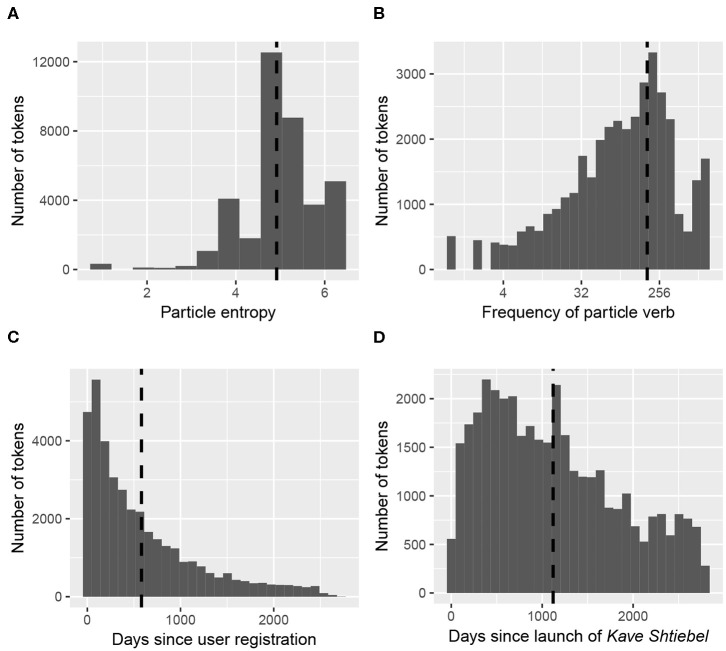
Raw distribution of particle verb tokens across significant continuous predictors (dashed lines represent the means; note that the x-axis of subplot B is on a logarithmic scale) **(A)** Particle entropy. **(B)** Frequency of particle verb. **(C)** Days since user registration. **(D)** Days since launch of *Kave Shtiebel*.

**Figure 5:**
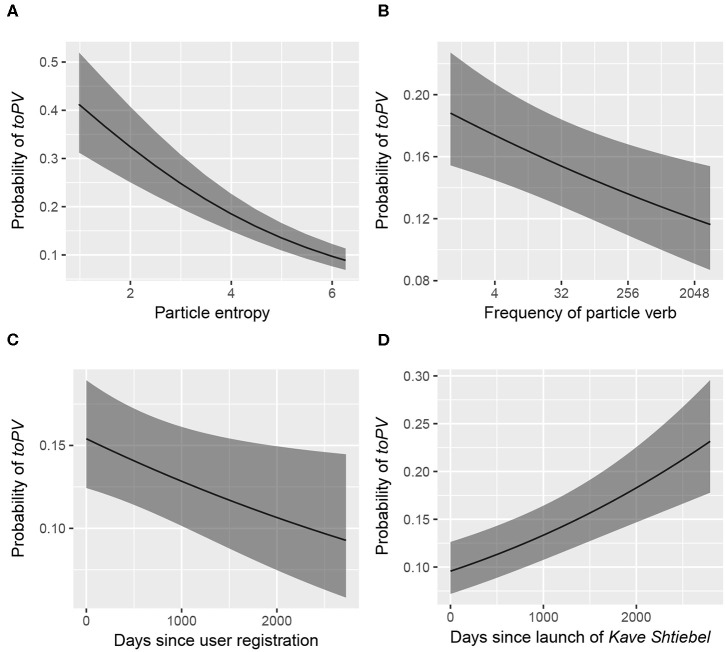
Predicted probability of *toPV* for significant continuous fixed effects (note that the x-axis of subplot B is on a logarithmic scale). **(A)** Particle entropy. **(B)** Frequency of particle verb. **(C)** Days since user registration. **(D)** Days since launch of *Kave Shtiebel*.

Since all continuous predictors were standardized (see their raw distributions in Figure 4), the estimates listed in Table 3 should be interpreted as follows: for every change of one standard deviation of a given effect, the log odds of the *toPV* variant increases (or decreases) by the estimate listed. Visualizations of the predicted effects are provided in Figure 5, showing how each of the significant continuous predictors relates to the predicted probability of *toPV*. For each subplot, the predicted probability of *toPV* is plotted at the average level of the other predictors in the model.

One of the more pronounced fixed effects is the number of days that have elapsed since the launch of KS: the more time that has passed (i.e., the more recent the post), the more likely the *toPV* variant is to be used. However, the number of days that have elapsed since user registration (i.e., the user's seniority as a KS member) has an overall *disfavoring* effect on the *toPV* variant. If *toPV* is being used relatively more often over time, then it seems paradoxical for writers to disfavor that variant the longer they interact on the forum. An in-depth discussion of these seemingly contradictory time effects is presented in section 5.2.

The other significant continuous fixed effects are particle entropy and the log frequency of the particle verb combination, which both pattern in ways consistent with the hypotheses outlined above. Particles with higher entropy disfavor the use of *toPV*, suggesting that particles that can more freely associate with different verbs (i.e., more productive particles) are also more tolerant of intervening *tsu* (*PtoV*). More frequent particle verb combinations favor the *PtoV* variant, which was expected under the assumption that high frequency combinations may have a more robust representation of the *PtoV* exemplar in episodic memory. Further investigation is needed in order to obtain a clearer picture of the role of frequency in constraining syntactic variation, in Yiddish and in other languages.

#### 5.1.2. Effects and Interpretations of Significant Categorical Predictors

**Figure 6:**
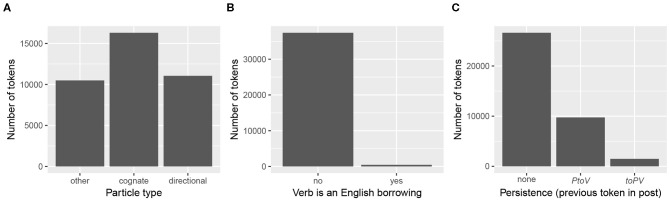
Raw distribution of particle verb tokens across significant categorical predictors. **(A)** Particle type. **(B)** Verb is an English borrowing. **(C)** Persistence (previous token in post).

**Figure 7:**
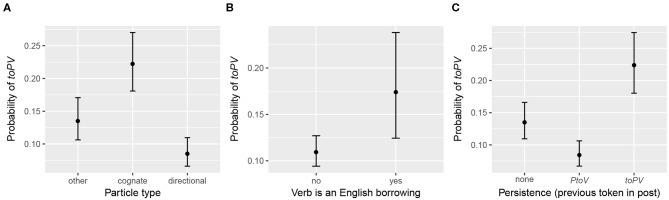
Predicted probability of *toPV* for significant categorical fixed effects. **(A)** Particle type. **(B)** Verb is an English borrowing. **(C)** Persistence (previous token in post).

The remaining significant fixed effects (particle type, whether the verb is an English borrowing, and variant persistence) are categorical variables. Their distributions are shown in Figure 6. Figure 7 plots the predicted marginal means, showing how each of the factor levels relates to the predicted probability of *toPV*. Again, for each factor, the predicted probability of *toPV* is plotted at the average level of the other predictors in the model.

Each of these categorical predictors has an effect on the variation in the direction hypothesized. Directional particles, which tend to contribute to the meaning of particle verb combinations in transparent or semantically compositional ways, tolerate the intervention of *tsu* (*PtoV*) at the highest rate. Cognate particles, which are often found in idiomatic or semantically non-compositional combinations, tolerate the intervention of *tsu* at the lowest rate (*toPV*). The “other” particles have an effect that is intermediate between the two types, and significantly different from both. There is a clear effect of whether the verb is an English borrowing, such that borrowed verbs favor *toPV* relative to other kinds of verbs. Note, however, that there is a massive imbalance across borrowings and non-borrowings (see Figure 6B), and consequently this effect should be interpreted with some caution. For example, for certain tokens tagged as having “English verbs,” it is actually the entire particle verb combination that is a borrowing, and in English the “particle” is actually an inseparable prefix (e.g., *op-deyt-n* ‘update’; cf. ^*^date up). These tokens understandably favor *toPV* (though never at 100%; e.g., there are 8 tokens of the *PtoV* variant *op-tsu-deyt-n* compared to 40 tokens of *tsu op-deyt-n*). Finally, there is a clear effect of persistence from the variant most recently used in the post, such that users are biased to repeat the same variant whether *PtoV* or *toPV*. Tokens of “none” are situated in the middle. This is to be expected, both because the absence of a previous token should not give rise to any persistence effect, and because the data are distributed in such a way that the majority of tokens are the first (or only token) of their respective posts (see Figure 6C). These findings lend themselves to follow-up analysis considering whether texts written for distribution on the internet (in Yiddish or any other language) generally exhibit stronger persistence effects than other genres of audience-oriented writing, in which the effects of cognitive constraints on variation may be tempered by more careful editing.

### 5.2. Discussion of Syntactic Change in Real Time

To reiterate one of the more intriguing findings of the statistical analysis, a seemingly contradictory effect was identified for the time elapsed since user registration and for the time elapsed since the launch of KS: users favor the standard *PtoV* variant the older their accounts are, despite a forum-wide trend favoring the non-standard *toPV* variant in real time. In other words, there seems to be evidence both for *individual change* toward greater use of *PtoV* and *community change* toward greater use of *toPV*.

#### 5.2.1. Implicit Standardization Favoring *PtoV* in Real Time

The finding that increased user seniority favors *PtoV* is consistent with the observation that online platforms, and KS in particular, have created new opportunities for Hasidic men to acquire experience and skill as Yiddish writers. In a sociolinguistic interview, one KS user Fayvl (31; Williamsburg) explicitly connected the advent of discussion forums to the proliferation of written standards:

*Kave Shtiebel* is trying to… the leaders of it, I don't know who they are, are trying to make Yiddish a, that it should have rules… It has changed quite a lot, actually. Because when I grew up, I mean, before the internet, there wasn't anywhere to write in Yiddish. A Hasid who wanted to write, he didn't have anywhere to write. You understand? Because…there just wasn't [any outlet]. Today you can write on the internet, or WhatsApp. We want to be able to write well. Automatically it's becoming a language, you know? The language is being formed from scratch, in a certain sense.

(Translated from Yiddish.)

Although the mention of “rules” here encompasses norms of spelling, punctuation, and vocabulary, Fayvl's view also offers a cogent explanation for the empirical finding that more experienced writers favor a conservative variant in syntax. The longer users spend on KS posting messages and interacting with other KS writers, the likelier it is that they will acquire the norms used by others, including grammatical norms.

**Figure 8:**
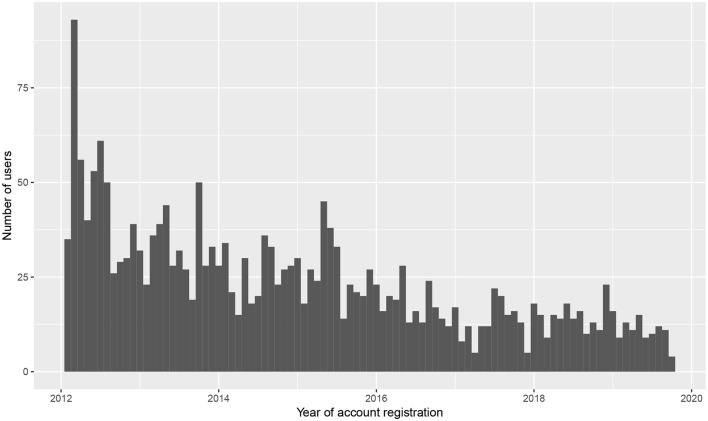
Users of *Kave Shtiebel* according to date of account registration (binned by month).

One of the distinct advantages of using a discussion forum as a linguistic corpus is that every post has a timestamp and every user has a registration date. This makes it trivial to organize users into cohorts and track their behavior over time—akin to a longitudinal panel study of spoken language across age cohorts. The approach pursued here is to group users based on year of account registration. Because the number of new KS users has stabilized since the forum's launch in 2012 (Figure 8), we collapse the most recent years (2015–now) into a single cohort.

**Figure 9:**
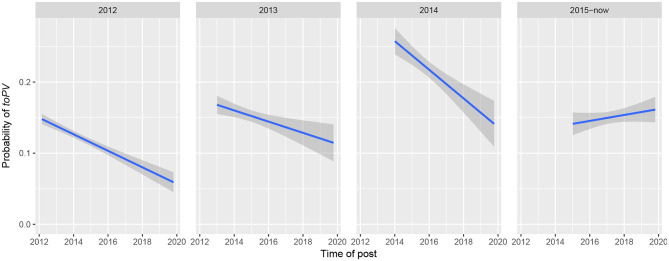
Regression lines showing the changing probability of *toPV*, based on plots of the raw distribution of tokens over time; data separated by the calendar year in which user registered on KS.

Figure 9 shows that for the largest single-year cohorts (2012, 2013, and 2014), who produced 81.7% of all tokens of non-finite particle verbs, users enter the forum with an increasingly high rate of *toPV*, which then falls over time. This suggests that regardless of when a cohort joins the forum, and regardless of what their initial rate of *toPV* is, by virtue of interacting with other users they seem to be acquiring the norm that associates *PtoV* with standard or “correct” usage. (The cohort since 2015 shows an increase in *toPV*, but the trend is flatter overall; if norms are being acquired implicitly, perhaps more time is required to see a decrease.)

Unlike inconsistencies in spelling, which are the object of explicit commentary online and offline, syntactic variation tends to fly under the radar of most writers. To my knowledge, there has been no discussion of the variation between *PtoV* and *toPV* on KS or any other Hasidic discussion forum. For this reason, and because the trend is observable even within single-year user cohorts, I take the finding about user seniority as empirical evidence of *rapid implicit standardization* among KS users.

If standardization is taking place on Hasidic social media more generally, the effect may actually be amplified on KS, where a writer's adherence to norms in spelling and punctuation is viewed as a sign that he is mature, intellectual, and worldly. These are qualities that are especially valued on KS, a forum that positions itself as challenging the Hasidic mainstream, particularly the perception of Hasidic “groupthink” which is so often criticized on the forum. Additional research using data from other forums could shed light on the factors motivating implicit standardization among Hasidic Yiddish writers.

#### 5.2.2. Community Change Favoring *toPV* in Real Time

If users favor the standard *PtoV* variant the longer their accounts remain open and active, it seems strange that there should also be a real-time effect favoring non-standard *toPV* on the forum overall. While it is possible that we are witnessing a genuine change in progress, one that reflects a possible increase in *toPV* in spoken Yiddish, it is surprising to find such an effect on a forum that has existed for under eight years, and whose users may not differ in age even if they joined the site at different times.

The contradiction is resolved if we acknowledge that there may be significant differences in the social characteristics of users depending on how recently they began writing on KS. As Figure 8 shows, a large number of users registered on KS within the first month or so of its launch. Because KS was founded as an offshoot of a different forum, *iVelt*, most of these early users already had a history of communicating in written Yiddish—certainly on *iVelt* if not on other online platforms, too. It stands to reason that these early users may have had a lower initial rate of *toPV* when KS first launched, since their development as Yiddish writers actually began elsewhere. (This is supported in Figure 9 by comparing the initial probability of *toPV* in the 2012 cohort against the subsequent cohorts from 2013 and 2014). If this view is correct, then a 36-year-old Hasidic Jew who registers on KS for the first time in 2019 may be much less experienced than a 36-year-old who joined KS seven years earlier. This could account for the conflicting trends in real-time data, where newcomers to the forum favor *toPV* even though individual users are expected to favor *PtoV* as they gain experience and facility with the norms of written Yiddish. Impressionistically, this explanation is supported by the fact that newcomers' welcome messages to the subforum *lomikh zikh forshteln far aykh* ‘let me introduce myself to you’ are substantially less standard in orthography and vocabulary than one finds among more senior writers. To test this explanation more directly, a follow-up study could compare the “standardness” of written Yiddish across different seniority levels on KS, in terms of users' grammatical norms as well as orthography and vocabulary.

## 6. Conclusions

While sociolinguists have acknowledged the hegemony of English in quantitative studies of variation, work on minority language varieties is still underrepresented (Meyerhoff and Nagy, 2008; Stanford, 2016; Guy and Adli, 2019). The shortage of research on these languages is especially pronounced in areas of linguistics where new computational methods have made it possible to identify complex trends in large messy datasets. As Nicholas Ostler has argued, “just as [the Yiddish philologist] Max Weinreich once remarked that a language is a dialect with an army and a navy, nowadays a language is a dialect with a dictionary, grammar, parser, and a multi-million-word corpus of texts, which are computer tractable, and ideally a speech database too” (Ostler, 2011, p. 320). As these computational resources continue to be developed in Hasidic Yiddish and other minority language varieties, corpus research will be able to uncover significant linguistic and social constraints on variability in a larger number of the world's languages.

This analysis of syntactic variation on a Hasidic Yiddish discussion forum has revealed that the choice of the *PtoV* or *toPV* order in non-finite particle verbs—seemingly arbitrary, given the presence of near-minimal pairs with equivalent semantics—is conditioned by both linguistic and social factors. The conditioning effects are also consistent with the findings from studies of particle verb variation in English. For example, the statistical analysis identified significant effects for particle type, which is taken to approximate the degree of semantic transparency, and for particle entropy, which is taken to approximate particle productivity across different verbs. Additional comparative studies are needed if variationists seek to evaluate the cross-linguistic applicability of conditioning factors assumed to be universal, e.g., the tendency to minimize syntactic and semantic dependencies (Lohse et al., 2004) or the tendency to repeat recent variants (Tamminga, 2016).

That some of the factors influencing particle verb variation in English also play a role in Hasidic Yiddish begs the question: Are these overlapping constraints due to universal linguistic properties, or is it possible that they arose in Yiddish due to contact with English? The latter hypothesis is consistent with an assumption widely held by Yiddish scholars and speakers alike, that *all* changes taking place in American Yiddish must ultimately derive from contact with English. In fact, some of the Hasidic men consulted during this project assured me that *toPV* is itself a structural borrowing from English, since *to* always comes before the verb in English. However, this explanation ignores the fact that *tsu* ‘to’ always precedes the verb in Yiddish as well, as shown in (3) for infinitives without particles.

In the absence of compelling evidence corroborating the English contact-based model, I maintain that the increased probability of *toPV* could be a Yiddish-internal development. First, although relatively rare, tokens of *toPV* can be found in pre-Holocaust Yiddish publications from Eastern Europe. In fact, some of the earliest examples of *toPV* come from traditional glosses of religious texts in Hebrew (Simon Neuberg, pers. comm.), such as Rashi's commentary on Genesis 14:9 *mi****lirdoyf****akhareyhem* ‘from chasing after them,’ glossed in Yiddish as *fun* ***tsu nokh yogn****zey* (lit., from **to after chase** them)^17^. Traditional Hebrew glossing, also known as *kheyder-taytsh* ‘school translation,’ often preserves the morpheme or word order of the Hebrew even if the resulting Yiddish is somewhat awkward structurally. The influence of such glosses on the development of Yiddish has been posited before (Timm, 2005), and it is plausible that the *l*-prefix marking Hebrew infinitives played some role in the emergence of *toPV*. The effect might be especially pronounced among Orthodox Jewish men, who were—and still are—exposed to such glosses in their *kheyder* education.

Second, separable particles never appear preverbally in English (*toVP*: *to throw up*; cf. *toPV* ^*^to up throw and *PtoV* ^*^up to throw), whereas particles invariably precede the verb in Yiddish infinitives. Third, the variation in English involves the relative ordering of particles and full noun phrase objects, and it is not limited to non-finite contexts (*I will call {up} the mayor {up}*; *I called {up} the mayor {up}*, etc.). In Yiddish, however, the relative ordering of particles and full noun phrase objects is generally fixed in the present tense, when verb-second (V2) movement causes the particle to appear postverbally:


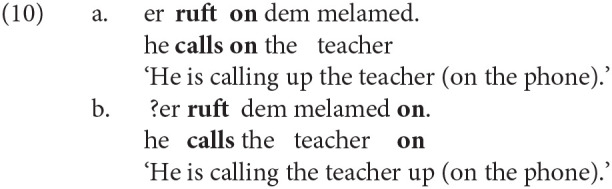


It is conceivable that Yiddish borrowed some of the underlying constraints on particle verb variation from English without borrowing its variant surface structures. However, it seems more plausible that the overlap in conditioning factors stems from language-independent considerations, which can be posited for all of the (non-social) predictors selected in the statistical model.

With respect to socio-stylistic constraints, the analysis revealed that a single online discussion forum can be a vehicle both for the spread of an innovative linguistic form and for the reinforcement of conservative written standards. This finding contributes to our understanding of the role that social media sites play in the rapid diffusion of linguistic change (e.g., Eisenstein et al., 2014). Given popular stereotypes about the internet as a place where language is “ruined”—where non-standard abbreviations, acronyms, and slang are spread—it is surprising that a discussion forum could be a venue for the proliferation of written norms. Perhaps *implicit* standardization is only possible in a language community that does not have a formal system for teaching and enforcing such written norms. Alternatively, implicit standardization could be a more general phenomenon affecting online writing, but researchers' focus on short-form media (such as text messages and tweets) has obscured this fact. Large corpus studies, especially of other minority language varieties, could shed light on this question of how language change occurs online, whether that change involves an increase or a decrease in the use of standard variants.

Finally, this study has demonstrated that robust patterns of language variation and change can be gleaned from a relatively modest online community of writers, using data drawn from posts written over a period of less than eight years. Even if the challenge of data scarcity looms large for machine translation in “low-resource” minority languages (Genzel et al., 2009)^18^, it should not deter sociolinguists from attempting to analyze variation in those languages. This result should inspire confidence that corpus sociolinguistics can uncover patterns of grammatical variation and change in minority language varieties, provided that specialists know where to find raw data and can define heuristics to identify tokens of variables. Studies of variation on social media platforms not only elucidate linguistic behavior on the internet, but they also generate testable hypotheses for research conducted in the speech community.

## Data Availability Statement

The datasets generated for this study are available on request to the corresponding author.

## Ethics Statement

The studies involving human participants were reviewed and approved by New York University, University Committee on Activities Involving Human Subjects. All participants provided their written informed consent.

## Author Contributions

The author confirms being the sole contributor of this work and has approved it for publication.

## Conflict of Interest

The author declares that the research was conducted in the absence of any commercial or financial relationships that could be construed as a potential conflict of interest.

## Footnotes

^1^As of November 2019, the first page of Google search results for the high-frequency trigram *vos iz der* ‘what is the.masc.sg’ includes a webpage entitled *vos iz der taytsh fun lebn?* ‘what is the meaning of life?’ from GotQuestions.org, an evangelical Christian missionary blog. Each page has been machine-translated into dozens of languages, and the Yiddish version is nearly incomprehensible.

^2^The largest annotated corpus available for any variety of Yiddish is the *Corpus of Modern Yiddish* (http://web-corpora.net/YNC/search/), a 4-million-word collection limited to texts published in the standardized YIVO orthography (YIVO, 1999), which is not used in any Hasidic community. By contrast, the *Kave Shtiebel* corpus assembled for this study contains approximately 29 million words from Hasidic Yiddish writers.

^3^*Katle kanye* is the most well-known Hasidic blogger, and his reputation extends far beyond KS. His self-published book on the problems of Hasidic education was recently recognized by the *Forward 50*: https://forward.com/series/forward-50/2018/katle-kanye/.

^4^There are a few lexicalized exceptions for which *toPV* is common (*iber-zets-n* ‘translate,’ *iber-tsayg-n* ‘convince,’ *iber-rash-n* ‘surprise,’ *unter-drik-n* ‘oppress’) in which the “particle” variably behaves like a prefix, so that it is not stressed and does not separate from the verbal root in the past participle or present tense conjugations. These exceptions are likely borrowings from Modern German, since *iber* ‘over’ and *unter* ‘under’ are not productive prefixes in Yiddish. Simon Neuberg (pers. comm.) has sent me examples of the *toPV* order that he has encountered in modern literary sources. Most of them come from Soviet writers whose native dialect was Northern (“Litvish”) Yiddish, which is geographically and linguistically distant from the Transcarpathian varieties considered to be the source of contemporary Hasidic Yiddish.

^5^I am grateful to Lea Schäfer and her student assistant Marc Brode, both of the *Syntax of Eastern Yiddish Dialects* (SEYD) project, for processing the relevant data from the *Language and Culture Atlas of Ashkenazic Jewry*. The *Atlas*'s survey includes question 020.020/021 ‘it's not nice *to stick out* your tongue,’ which explicitly targets the acceptability of *toPV*. The only informants who supplied or accepted *toPV* came from cities and towns in present-day Belarus, Lithuania, and Eastern Poland. Transcarpathian speakers rejected *toPV*.

^6^Even in the longest sociolinguistic interviews I conducted in the New York area, it is difficult to find more than five tokens of non-finite particle verbs per speaker.

^7^The use of a different set of labels for the variants in Yiddish (*toPV* and *PtoV*, rather than “continuous” and “discontinuous”) is justified for several reasons. First, the English variants both have postverbal particles, while the Yiddish variants both have preverbal particles. Second, in English the continuous variant is evaluated as more normatively “correct” than the discontinuous variant, while in Yiddish the “discontinuous” or nonadjacent variant (*PtoV*) is preferred. Finally, using new labels minimizes the confusion likely to arise when referring to a “continuous variant” in syntax and a “continuous variable” in statistical analysis.

^8^Mark (1978) and others have observed that the non-directional particles often contribute some aspectual meaning to the verb (as in English *eat* vs. *eat up*). See also Gold, 1999, chapter 1.

^9^The entropy of a particle is defined as the negative sum of the probability of each unique verb that occurs with that particle multiplied by the log probability of that same verb, i.e., $H\left(\text{particle}\right)=-\underset{\text{v}\in \text{verbs}}{\sum }p\left(\text{v}\right)\times \log_{2}p\left(\text{v}\right)$. Its implementation in Python is given in Bird et al., 2009, example 6–8.

^10^The standard ideology motivating this difference is unclear. The continuous order may be favored if speakers are reanalyzing particles as prepositions, which according to the norms of standard usage should not appear in sentence-final position.

^11^The removal of final forms (and apostrophes) makes it easier to recognize variant spellings as instances of a single lexical item. For example, verbs with roots derived from Hebrew, e.g., *kholem-en* dream(Hebrew)-INF(Germanic) ‘dream,’ are inconsistently spelled with final letters (and apostrophes) before the Germanic infinitival suffix. The stripping of final forms in Yiddish is somewhat analogous to converting capital letters to lowercase in English.

^12^Three particles were omitted in order to avoid false positives and categorization errors: *tsu*, which coincides with the non-finite tense marker ‘to’ and therefore makes the *PtoV* and *toPV* orders indistinguishable; *for*, which is spelled just like the (inseparable) verbal prefix *far*; and *um*, which coincides with the adjectival prefix meaning ‘un-.’

^13^Examples include variants that reflect regional pronunciations (e.g., *inter* for *unter* ‘under’) and reduced forms (e.g., *tsam*, equivalent to standard *tsuzamen* ‘together’).

^14^A silent letter *alef* is used in Yiddish to break up three adjacent repeated letters (the so-called *mekhitse-alef* ‘barrier *alef*’; Katz, 1993, p. 139), as when a single *vov* representing [u] appears in front of a double *vov* representing [v]: *aroys-ts****u****-*alef*-****v****ayz-n* outward-to-show-INF ‘to display.’ All *mekhitse-alef* s at the beginning of verb strings were manually removed.

^15^For example, the single letter ‘*g*’ appeared in the list of verbs. This is because, purely by coincidence, the plural noun *oystsugn* ‘excerpts’ looks like it begins with the particle *oys* ‘out,’ followed by *tsu*, and ending with -*n* (which is also the plural suffix). “*G*” is not a verb (or a word) and was removed from the list.

^16^Because non-finite particle verbs can theoretically appear as one, two, or three separate words with spaces, only the following forms were counted as tokens: one word (*PtoV* or *toPV*), two words (*to PV*), or three words (*to P V*). The other possible spacing patterns were excluded to avoid false positives.

^17^This particular example was found in a Yiddish edition of the *mikroes-gdoyles* (the Hebrew Bible along with various commentaries) published in Vilnius in 1899 but which may be based on an older translation. Available online: https://books.google.com/books?id=7W4_AAAAYAAJ, p. 122.

^18^Thanks to improvements in optical character recognition, the Yiddish Book Center now supports text search across its collection of over 10,000 volumes (ocr.yiddishbookcenter.org). This could be a boon for machine translation in Yiddish.
